# Presynaptic NMDA Receptors Mediate IPSC Potentiation at GABAergic Synapses in Developing Rat Neocortex

**DOI:** 10.1371/journal.pone.0017311

**Published:** 2011-02-18

**Authors:** Seena S. Mathew, John J. Hablitz

**Affiliations:** Department of Neurobiology and Evelyn F. McKnight Brain Institute, University of Alabama at Birmingham, Birmingham, Alabama, United States of America; Institut National de la Santé et de la Recherche Médicale, France

## Abstract

**Background:**

NMDA receptors are traditionally viewed as being located postsynaptically, at both synaptic and extrasynaptic locations. However, both anatomical and physiological studies have indicated the presence of NMDA receptors located presynaptically. Physiological studies of presynaptic NMDA receptors on neocortical GABAergic terminals and their possible role in synaptic plasticity are lacking.

**Methodology/Principal Findings:**

We report here that presynaptic NMDA receptors are present on GABAergic terminals in developing (postnatal day (PND) 12-15) but not older (PND21-25) rat frontal cortex. Using MK-801 in the recording pipette to block postsynaptic NMDA receptors, evoked and miniature IPSCs were recorded in layer II/III pyramidal cells in the presence of AMPA/KA receptor antagonists. Bath application of NMDA or NMDA receptor antagonists produced increases and decreases in mIPSC frequency, respectively. Physiologically patterned stimulation (10 bursts of 10 stimuli at 25 Hz delivered at 1.25 Hz) induced potentiation at inhibitory synapses in PND12-15 animals. This consisted of an initial rapid, large increase in IPSC amplitude followed by a significant but smaller persistent increase. Similar changes were not observed in PND21-25 animals. When 20 mM BAPTA was included in the recording pipette, potentiation was still observed in the PND12-15 group indicating that postsynaptic increases in calcium were not required. Potentiation was not observed when patterned stimulation was given in the presence of D-APV or the NR2B subunit antagonist Ro25-6981.

**Conclusions/Significance:**

The present results indicate that presynaptic NMDA receptors modulate GABA release onto neocortical pyramidal cells. Presynaptic NR2B subunit containing NMDA receptors are also involved in potentiation at developing GABAergic synapses in rat frontal cortex. Modulation of inhibitory GABAergic synapses by presynaptic NMDA receptors may be important for proper functioning of local cortical networks during development.

## Introduction

GABA is the principal inhibitory neurotransmitter in the neocortex. Responses mediated by GABA_A_ receptors are already present embryonically [1] and regulate excitability during postnatal development of the neocortex [2]. Formation of local neocortical circuits is dependent upon electrical activity, both spontaneous and experience driven [3]. GABA is known to shape circuit function by controlling activity-dependent refinement of functional connections [4] and by changing the balance between excitation and inhibition [5]. Plasticity at GABAergic connections also has been implicated in the establishment of synaptic networks [6].

NMDA receptors are tetrameric ionotropic glutamate receptors that consist of two obligatory (NR1) and two regulatory subunits, either NR2 or NR3. NMDA receptors are traditionally viewed as being located postsynaptically, at both synaptic and extrasynaptic locations. However, both anatomical [7]–[8] and physiological studies [9]–[11] have indicated the presence of NMDA receptors located presynaptically. In entorhinal cortex, when postsynaptic NMDA receptors were blocked by intracellular MK-801, bath application of D-APV decreased the frequency of miniature (m) EPSCs indicating that NMDA receptors tonically facilitated transmitter release [11]. Similar decreases in mEPSC frequency were observed with the NR2B subunit-specific antagonist ifenprodil, indicating involvement of NR2B-containing receptors [12]. Functional presynaptic NMDA receptors have also been reported in visual cortex where NR2B containing receptors again appeared to be involved (see review by [13]). Presynaptic NMDA receptor expression in cortical structures is developmentally regulated [14]-[15] and down regulation has significant effects on synaptic plasticity [15].

Electron-microscopic studies have demonstrated presynaptic NMDA receptors on GABAergic nerve terminals in cerebellum and neocortex [16]–[17]. Activation of presynaptic NMDA receptors increases the frequency of spontaneous and mIPSCs in cerebellar basket, stellate and Purkinje cells [18]–[19]. Long-term potentiation (LTP) of GABAergic synapses, via a presynaptic mechanism, has been reported at synapses in the cerebellum [20] and ventral tegmental area [21]. Physiological studies of presynaptic NMDA receptors on neocortical GABAergic terminals and their possible role in synaptic plasticity are lacking. Our results indicate that presynaptic NMDA receptors can tonically modulate GABA release in neocortex and that physiologically patterned stimulation can induce synaptic plasticity in inhibitory currents in pyramidal cells. These modulatory effects are developmentally regulated, involve NR2B subunit containing receptors and may influence development of neocortical circuits.

## Results

### Presynaptic NMDA receptors facilitate inhibitory synaptic transmission in immature neocortex

**Figure 1:**
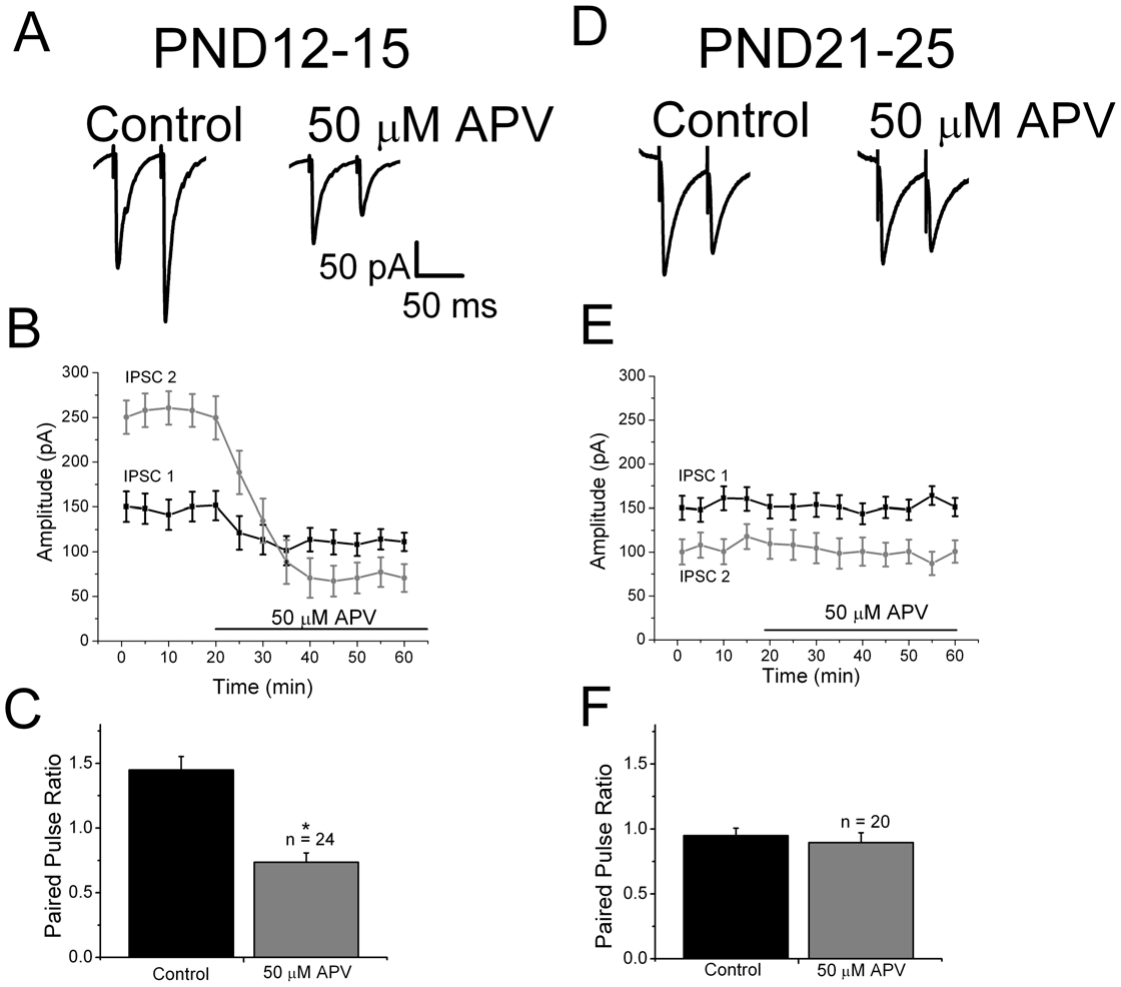
Physiological evidence for modulation of GABAergic IPSCs by presynaptic NMDA receptors in rat neocortex. *A:* in slices from PND12-15 animals, paired pulse facilitation was observed under control conditions (left trace). After bath application of D-APV, the same stimulation evoked a smaller IPSC 1 and subsequent paired pulse depression. These results suggest the presence of tonically activated facilitatory presynaptic NMDA receptors. *B:* time course plot of IPSC 1 and IPSC 2 amplitudes showing effect of D-APV on each response. *C:* summary plots show the effect of D-APV on paired pulse ratios in PND12-25 group. *D:* specimen records from PND21-25 animals showing paired pulse depression under control conditions. Responses did not change after wash in of APV. *E:* time course plot of IPSC 1 and IPSC 2 amplitudes showing that D-APV had no effect on either response in the PND21-25 group. *F:* summary plots show developmental changes in the effect of APV on paired pulse ratios. IPSCs were pharmacologically isolated by recording in the presence of 50 µM GYKI52466, 20 µM CNQX and 2 µM SCH50911 to block AMPA, KA and GABA_B_ receptors, respectively. The recording pipette contained 1 mM MK-801 to block postsynaptic NMDA receptors. Specimen records shown are averages of 10 responses.

Modulation of inhibitory transmission in neocortex by presynaptic NMDA receptors, and possible developmental regulation, has not been examined. The effect of presynaptic NMDA receptors on evoked GABA release was studied by recording pharmacologically isolated IPSCs in the presence of 50 µM GYKI52466, 20 µM CNQX and 2 µM SCH50911 to block AMPA, KA and GABA_B_ receptors, respectively. We have shown previously that currents evoked under these conditions were blocked by 10 µM bicuculline, a GABA_A_ receptor antagonist, suggesting that the observed currents were mediated through activation of GABA_A_ receptors [22]. Recordings were obtained from layer II/III pyramidal cells. Contributions from postsynaptic NMDA receptors were blocked by including 1 mM MK-801 in the pipette solution and voltage clamping the cell at −70 mV in the presence of 1.3 mM Mg^2+^ [11], [15]. As shown in Fig. 1A, when a pair of stimuli was applied at an interstimulus interval of 50 ms, paired pulse facilitation was observed. This was seen in all PND12-15 neurons tested (n = 24). After obtaining control recordings, the competitive NMDA receptor antagonist D-APV (50 µM) was bath applied. The amplitude of both IPSC 1 and IPSC 2 were significantly reduced in the presence of D-APV (IPSC 1 from 155±0.02 pA to 114±0.3 pA, p<0.05 and IPSC 2 from 250±0.04 to 77±0.03 pA test, p<0.05, n = 24). A time course plot showing the effect of D-APV on IPSC 1 and IPSC 2 is shown in Fig. 1B. Paired pulse facilitation was changed to depression after D-APV application (Fig. 1C). The ratio changed from 1.4±0.1 to 0.7±0.1, p<0.05 in the presence of D-APV. The change in the first IPSC amplitude can be attributed to a D-APV-induced block of a tonic presynaptic NMDA facilitatory effect produced by ambient glutamate [23].

A developmental decrease in the ability of presynaptic NMDA receptors to facilitate glutamate release in mouse visual cortex has been reported previously [15]. To examine if a similar decrease of presynaptic NMDA receptors also occurred with IPSCs, we recorded from PND21-25 animals. As shown in Fig. 1D, PND21-25 animals showed paired pulse depression under control conditions, as reported in adult neocortex [24]. In PND21-25 animals, bath application of D-APV did not significantly affect the amplitudes of IPSC 1 or IPSC 2 (Fig. 1E) or paired pulse ratios (0.9+/−0.1, control; 0.89+/−0.07, APV, p>0.05) (Fig. 1F). These results suggest a decrease in facilitatory presynaptic NMDA receptors with development.

### Effects of NMDA receptor agonists and antagonists on mIPSCs

**Figure 2:**
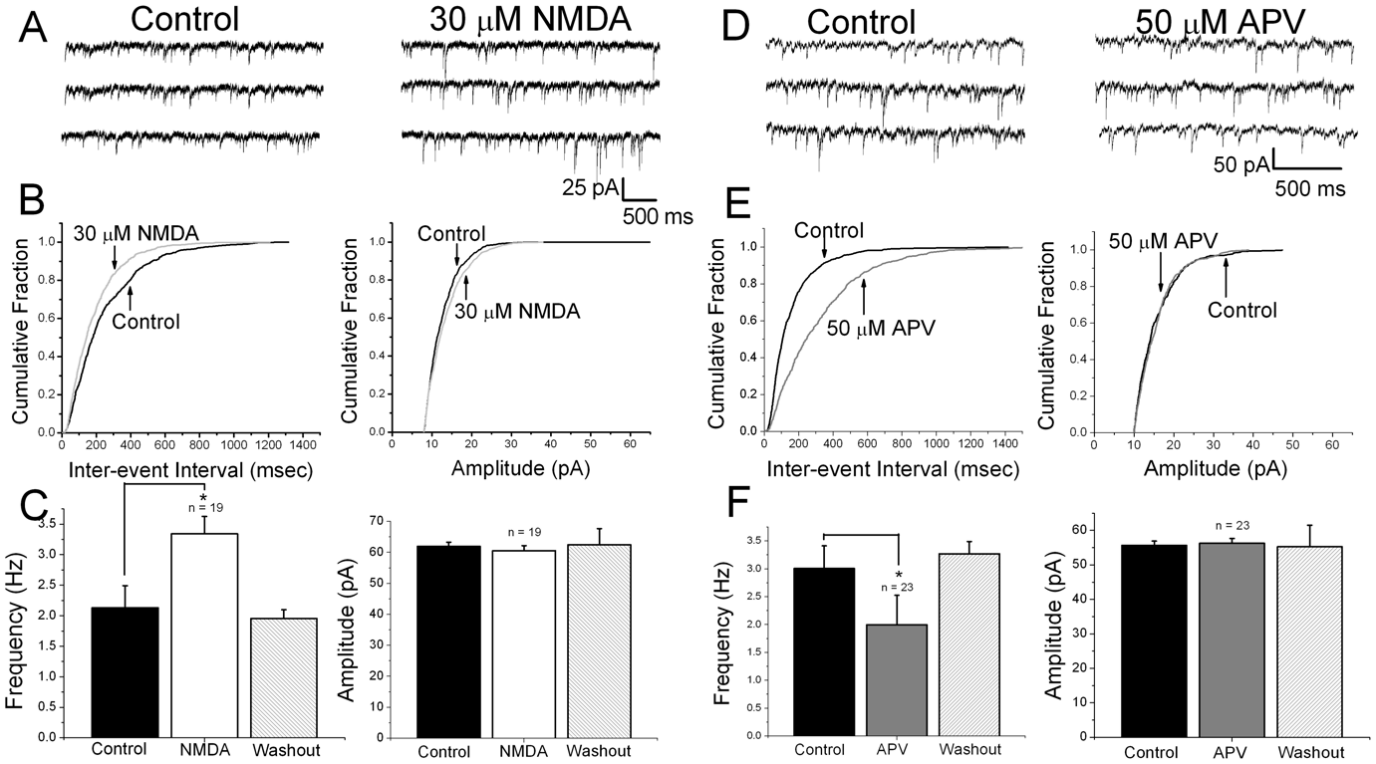
Effects of activation and inhibition of presynaptic NMDA receptors on mIPSC frequency in slices from PND12-15 rats. *A:* representative traces under control conditions (left) and after (right) bath application of 30 µM NMDA. *B:* cumulative probability plots of mIPSC intervals for the neuron shown in A (left) show that application of NMDA in the presence of 1 µM TTX decreased inter-event intervals, as indicated by the leftward shift in the presence of NMDA. There was no change in mIPSC amplitude distributions for this neuron after NMDA application (right). *C:* summary graphs show that bath application of NMDA significantly increased the frequency of mIPSCs without affecting the amplitude. *D:* representative traces under control conditions (left) and after bath application of 50 µM D-APV (right). A clear decrease in mIPSC frequency in the presence of APV was observed. *E:* cumulative probability plots of mIPSCs show that application of APV in the presence of 1 µM TTX increased the inter-event interval between mIPSCs. This is indicated by the rightward shift of the curve in the presence of D-APV (left). No change in mIPSC amplitude with D-APV application was observed (right). *F:* summary graphs show that bath application of APV significantly decreased the frequency of mIPSCs without affecting the amplitude. The effects on frequency were reversed upon washout of D-APV.

The results on evoked IPSCs and paired pulse ratios described above suggest modulation by presynaptic NMDA receptors. To further address presynaptic effects, pharmacologically isolated mIPSCs were recorded in the presence of 50 µM GYKI52466, 20 µM CNQX, 2 µM SCH50911 and 1 µM TTX to block AMPA, KA, GABA_B_ receptors and action potentials, respectively. A pipette recording solution to which 1 mM MK-801 had been added was used to block postsynaptic NMDA receptors [11]. It was hypothesized that if presynaptic NMDA receptors were present on GABAergic nerve terminals, bath application of NMDA would increase the frequency of mIPSCs without affecting amplitudes in PND12-15 animals. Conversely, NMDA receptor antagonists would decrease the frequency of mIPSCs with no change in amplitude. Specimen records of mIPSCs in a PND12 animal under control conditions are shown in Fig. 2A, left. After bath application of 30 µM NMDA to activate NMDA receptors, an increase in frequency was clearly observable (Fig. 2A, right). Cumulative probability plots of mIPSC interevent intervals and amplitudes for the neuron shown in Fig. 2A are shown in Fig. 2B, left and right, respectively. NMDA application shifted the interevent interval curve to the left indicating an increase in frequency whereas the amplitude plots were unaffected. A summary of results with NMDA application is shown in Fig. 2C. mIPSC frequency was significantly increased (2.1±0.4 control versus 3.6±0.3 Hz NMDA, n = 19, p<0.05) with no change in amplitude (61.9±1.3 control versus 58.6±4.0 pA NMDA, n = 19, p>0.05). These effects were reversible upon washing (Fig. 2C right). In these experiments, no change in holding current was observed with NMDA application demonstrating that postsynaptic NMDA receptors were blocked by the MK801 present in the recording pipette.

In the next series of experiments, 50 µM D-APV was bath applied to block NMDA receptor activation. Inspection of specimen records from a PND15 animal indicated that D-APV produced a decrease in the frequency of mIPSCs which was reversible upon washing (Fig. 2F). This occurred without a change in the amplitude (Fig. 2D, control versus APV). Fig. 2E, left and right, shows cumulative probability plots for interevent intervals and amplitudes, respectively. D-APV shifted the interevent interval plot to the right, indicating a decrease in frequency whereas amplitudes plots were superimposable. Fig. 2F summarizes the results from all cells tested. D-APV significantly decreased mIPSC frequency (3.0±0.4 control versus 2.0±0.5 Hz APV, n = 23, p<0.05) whereas amplitudes were unaffected (55.6±1.3 control versus 56.2±1.4 pA APV, n = 23, p>0.05). These results indicate that, in PND12-15 animals, presynaptic NMDA receptors are tonically active and facilitate GABA release.

**Figure 3:**
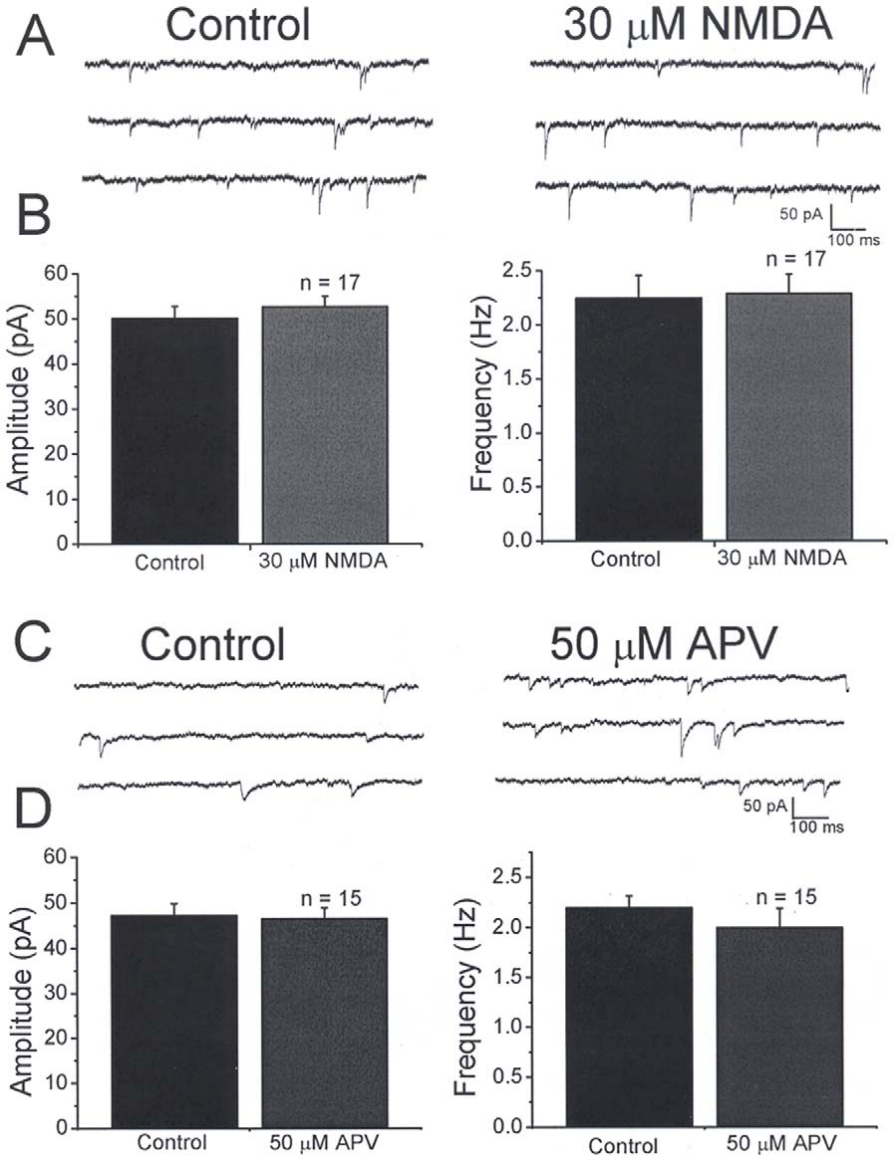
Miniature IPSCs in PND21-25 rats are not affected by bath application of NMDA or D-APV. *A:* representative traces under control conditions (left) and after bath application of 30 µM NMDA (right). No changes in mIPSCs were observed. B: bar graphs showing that bath application of NMDA did not have any significant effect on mIPSC amplitude (left) or frequency (right). *C:* representative traces under control conditions (left) and after bath application of 50 µM APV (right). *D:* bar graphs showing that bath application of APV did not affect the frequency or the amplitude of mIPSCs. This suggests that presynaptic NMDA receptors do not modulate GABA release in PND 21-25 rats.

Neurons from PND21-25 control rats were also tested. Fig. 3A shows specimen records under control conditions and in the presence of 30 µM NMDA. Agonist application did not have any obvious effect on mIPSCs. Quantitative analysis of mIPSC frequency and amplitude did not detect any significant differences, as shown in Fig. 3B. IPSC amplitudes were 50.2±2.5 and 52.6±2.3 pA (n = 17, p<0.05) before and after NMDA respectively. The frequency of IPSCs was 2.2±0.2 and 2.3±0.1 (n = 17, p<0.05) under control conditions and after NMDA. Similarly, antagonism of NMDA receptors with D-APV did not produce significant changes in mIPSC frequency or amplitude (Fig. 3, C and D). IPSC amplitudes were 47.2±2.6 and 46.5±2.4 pA (n = 15, p<0.05) before and after D-APV. The frequency of IPSCs was 2.3±0.1 and 1.9±0.3 (n = 15, p<0.05) under control conditions and after D-APV. Taken together, these results indicate that presynaptic NMDA receptors on inhibitory terminals appear to be downregulated by PND21-25.

### Presynaptic NMDA receptors mediate IPSC plasticity

**Figure 4:**
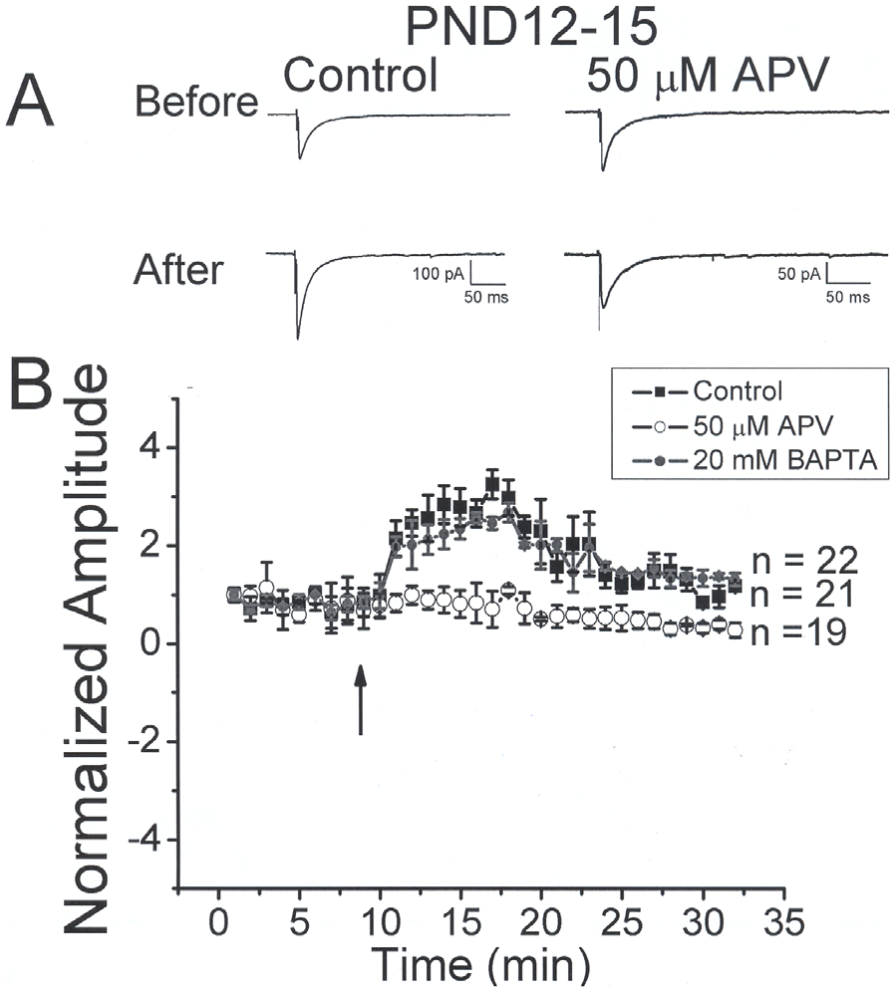
Patterned stimulation (10 bursts at 1.25 Hz with each burst consisting of 10 stimuli at 25 Hz) induces long term potentiation of IPSCs in neocortical slices from PND12-15 animals. *A:* specimen records showing representative traces before and after stimulation under control conditions (left) and in the presence of APV (right). Response enhancement was not observed in the presence of APV. *B:* summary plot showing time course plots of IPSC amplitude before and after patterned stimulation. LTP was seen in control and BAPTA groups but not in the presence of APV.

To further evaluate the role of presynaptic NMDA receptors in regulating synaptic plasticity at inhibitory terminals in neocortex, we examined the effects of patterned stimulation on evoked IPSCs. To minimize contribution from postsynaptic NMDA receptors, neurons were held at −70 mV in the presence of 1.3 mM Mg^2+^ and with MK-801 in the recording pipette. Under these conditions, ten episodes of patterned stimulation [25] were applied in slices from PND12-15 animals. Specimen records of averaged control IPSCs before and after patterned stimulation are shown in Fig. 4 A (left). During a ten minute baseline period, stimulation at 0.05 HZ produced IPSCs of constant amplitude, as shown in Fig. 4B. Under control conditions, stimulation produced a 178±0.4% (n = 21, p<0.05) increase in IPSC amplitude after 5 minutes. A stable significant increase of 35±0.1% (P<0.05 compared to pre stimulation baseline) was observed from 21 to 29 min after patterned stimulation. At many synapses, LTP mediated by postsynaptic NMDA receptors is blocked by including the calcium chealtor BAPTA in the recording pipette. We reasoned that if IPSC LTP was mediated by presynaptic NMDA receptors, postsynaptic BAPTA would not block LTP induction. After obtaining a stable whole cell recording, at least 15 min was allowed for BAPTA to diffuse into the cell. Fig. 4B shows that robust LTP was observed when neurons were loaded with 20 mM BAPTA. IPSC amplitudes were increased 134±0.2% (n = 22, p<0.05) 5 min after patterned stimulation. A stable significant increase of 36+/−0.1% (p<0.05) was observed from 21 to 29 min. Our results with evoked IPSCs and mIPSCs indicate that modulation by presynaptic NMDA receptors is blocked by application of D-APV. We hypothesized that D-APV also should block stimulation induced increases in IPSC amplitude if presynaptic NMDA receptors were involved, given that postsynaptic receptors were already blocked under our recording conditions. Specimen records of averaged IPSCs are shown in Fig. 4A (right). When stimulation was applied in the presence of 50 µM D-APV, LTP was not present at 5 min (19±0.2%, n = 19, p<0.05) (Fig. 4B right). A late depression of 56±0.1% (p>0.05) was observed from 21 to 29 min after stimulation, similar to that described for excitatory responses in the dentate gyrus of young rats [26]. These results suggest that, in PND12-15 animals, presynaptic NMDA receptors are necessary for patterned stimulation-induced IPSC LTP.

**Figure 5:**
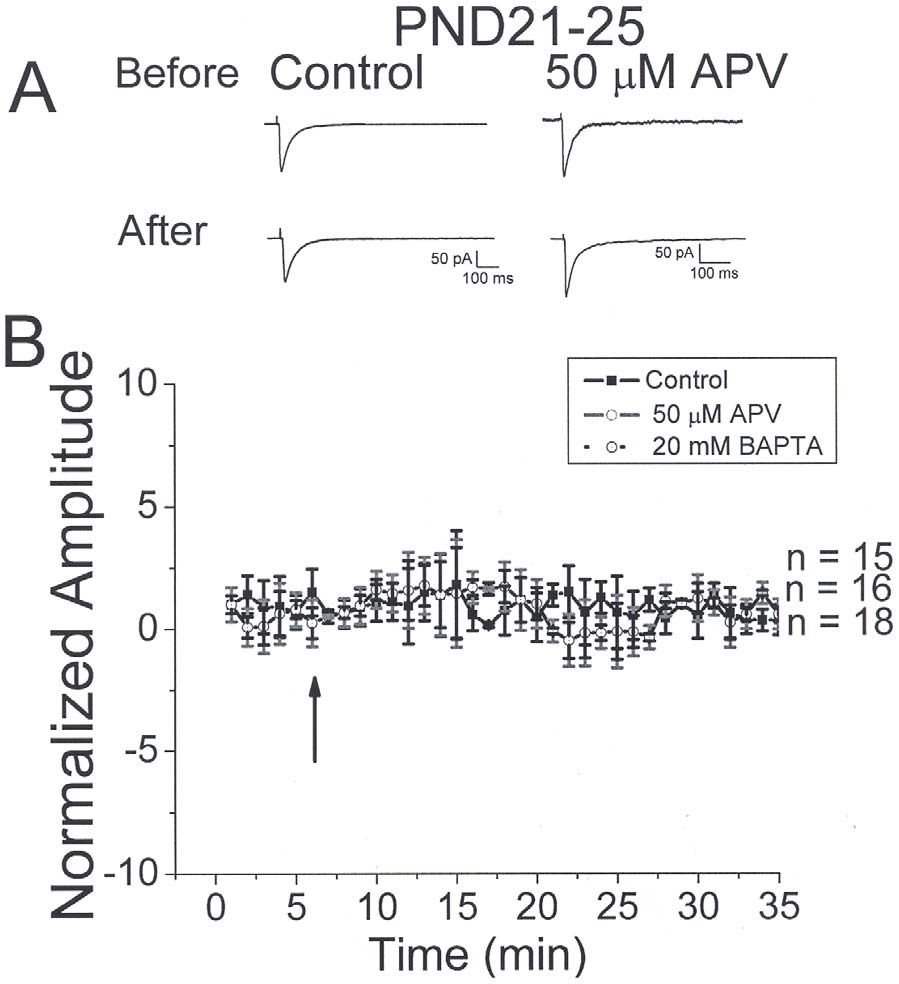
IPSC potentiation is not seen following patterned stimulation in PND21-25 animals. *A:* specimen records showing representative traces before and after stimulation under control conditions (left) and in the presence of APV (right). Response enhancement was not seen in either group. *B:* summary plot showing of IPSC amplitude before and after stimulation. LTP was not seen in control, BAPTA or APV groups.

Our present results with IPSCs and previous reports on EPSCs [11], [15] indicate that presynaptic NMDA receptors are downregulated by PND21-25. We hypothesized that if this were true, patterned stimulation would not induce LTP in this age group. To examine this, recordings were again obtained at −70 mV in the presence of 1.3 mM extracellular Mg^2+^, AMPA, KA and GABA_B_ antagonists in the bath and MK801 in the pipette. Representative recordings before and after patterned stimulation are shown in Fig. 5A (left). Under control conditions, no LTP was observed (n = 18). When additional experiments were performed in the presence of D-APV or BAPTA, LTP was also not observed (n = 16 and 18, respectively). Summary histograms of IPSC amplitudes before and after patterned stimulation are shown in Fig. 5B. Patterned stimulation did not have a significant effect on IPSC amplitude in the PND21-25 age group under any experimental condition.

NR2B-containing presynaptic NMDA receptors participate in tonic facilitation of glutamate release in epileptic rats [14] and LTD induction in layer V pyramidal cells [27]. We tested the hypothesis that NR2B-containing receptors contribute to IPSC plasticity by examining the effects of the NR2B subunit selective antagonist Ro 25-6981 on mIPSCs and stimulation induced changes.

**Figure 6:**
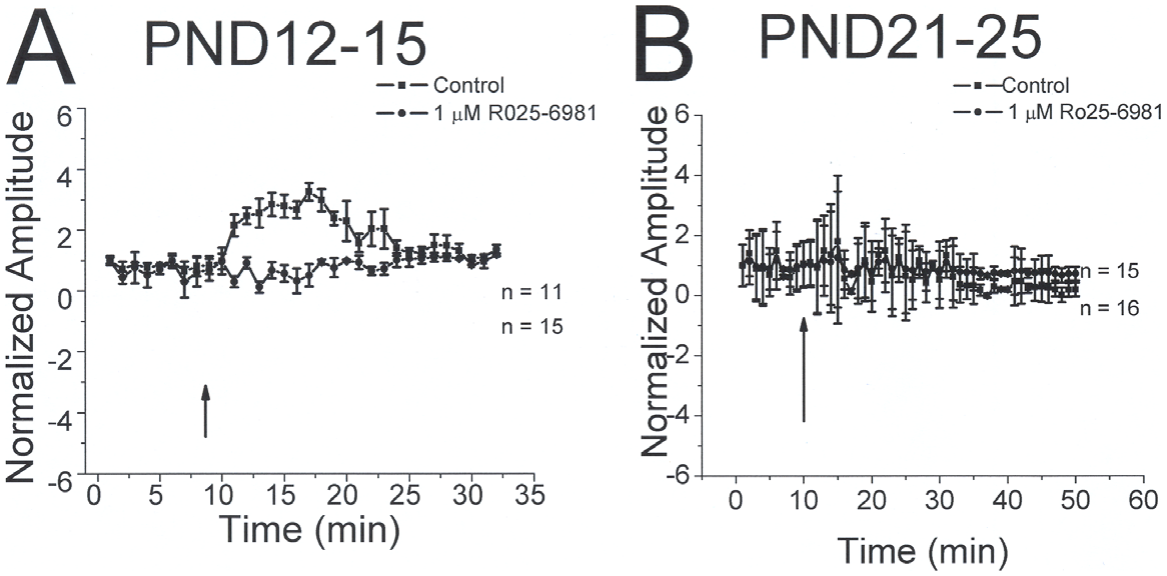
The NR2B subunit specific antagonist R025-6981 blocks IPSC potentiation in PND12-15 animals. A: Patterned stimulation induces long term potentiation of IPSCs in neocortical slices from PND12-15 animals. *A:* summary plot showing time course plots of IPSC amplitude before and after patterned stimulation. LTP was seen in control group but not in the presence of R025-6981. B: In PND21-25 group, potentiation was not seen in control or Ro25-6981 conditions.

As shown in Fig. 6A, under control conditions In PND12-15 rats, there was a 226±18% (n = 11, p<0.05) increase in IPSP amplitude 5 min after stimulation. In the presence of 1 µM Ro 25-6981 IPSP amplitude was not significantly changed 5 min following stimulation (2±1.7% decrease, n = 15, p>0.05). In PND21-25 animals, stimulation did not induce LTP (n = 15) and responses were unaffected by R0 25-6981 (n = 16) (Fig. 6B).

## Discussion

The present results indicate that presynaptic NMDA receptors modulate GABA release onto neocortical pyramidal cells. We provide evidence for tonic modulation of inhibitory synaptic transmission via activation of presynaptic NMDA receptors by ambient glutamate. Patterned stimulation-induced LTP of inhibitory synaptic transmission was dependent on activation of pre- but not postsynaptic NMDA receptors. These effects are developmentally regulated, being prominently displayed at PND12-15 but absent at PND21-25. Modulation of inhibitory GABAergic synapses by presynaptic NMDA receptors may be important for proper functioning of local cortical networks during development.

### Tonic activation of presynaptic NMDA receptors

Tonic regulation of synaptic glutamate release by presynaptic NMDA receptors has been reported previously in entorhinal [11]–[12] and neocortex [13]. Bath application of NMDA receptor antagonists, including the NR2B-subunit selective antagonists ifenprodil and Ro25-6981, consistently reduced the frequency but not amplitude of mEPSCs. Presumably, activation of presynaptic NMDA receptors by ambient levels of glutamate produces a tonic elevation of mEPSC frequency. Our previous studies have shown that ambient glutamate concentrations in neocortical brain slices are high enough to activate postsynaptic NMDA [28] and presynaptic kainate receptors [29]. Ambient extracellular glutamate levels have been measured using microdialysis, push-pull perfusion and electrochemical sensors, yielding estimates in the range of 0.5 to 5 uM [30]. Physiological studies estimating glutamate levels in hippocampal brain slices have indicated levels in the nanomolar range [31]. Ambient glutamate levels were not dependent on activity levels or vesicular release, leading to the suggestion that it is glial in origin [31]. These studies in large part use extrasynaptic NMDA receptors as glutamate detectors and do not address possible compartmentalization of glutamate levels in synaptic or perisynaptic regions. Pharmacological studies of changes in mEPSC frequency in response to NMDA [11], [15] and mGluR [32] antagonists clearly support the concept that ambient glutamate concentrations are sufficiently high to modulate glutamate release. Evidence for tonic activation of presynaptic NMDA receptors also comes from paired recording studies of neocortical neurons showing that EPSC amplitudes were reduced by APV. Analysis of coefficient of variation and paired pulse ratios indicated involvement of a presynaptic mechanism [27].

The exact location of the presynaptic NMDA receptors modulating glutamate transmission has not been established. It has been suggested that NMDA receptors expressed on the soma, dendrites or surrounding cells, rather than in axonal compartments might mediate observed changes in glutamate release [33], [34]. NMDA mediated depolarizations and Ca^2+^ entry in L5 pyramidal cell axons were not observed following focal application of NMDA receptor agonists [34]. GABAergic interneurons have not been similarly investigated. Changes in axonal excitability could be tested using whole cell recording from GABAergic interneurons and examining changes in threshold for eliciting antidromic spikes [35]. Evidence from changes in mIPSCs and lack of postsynaptic responses to bath applied NMDA suggest that the effects seen here are pre- not post-synaptically mediated, as. The exact location of the presynaptic effect (synaptic terminal, axon or dendrite) remains to be determined.

GABA receptors can also be activated by ambient concentrations of neurotransmitters. Ambient levels of GABA activate postsynaptic GABA_A_-receptors resulting in tonic inhibition [36] whereas activation of presynaptic GABA_B_-receptors modulates phasic inhibition [37]. Presynaptic ionotropic GABA receptors also modulate GABA release [38]. We have previously reported that ambient glutamate levels activate kainate receptors located on GABAergic terminals producing facilitation of GABA release in the neocortex [22]. In the present study, the changes in evoked IPSC amplitude following application of NMDA receptor antagonists suggest similar tonic facilitation of GABA release by presynaptic NMDA receptors. This tonic modulation via NMDA receptors does not result in maximal potentiation since bath application of NMDA can further increase mIPSC frequency. Regulation of GABA release thus appears to be controlled by glutamate via a variety of modulatory systems.

### IPSC potentiation and NR2B receptors in developing neocortex

Potentiation at excitatory synapses has been extensively studied. Considerable evidence also indicates that long term changes in excitability of GABA-mediated synaptic transmission occur. LTP of GABAergic synapses has been reported in CA1 pyramidal cells following tetanic stimulation [39]. High frequency stimulation has also been shown to produce IPSC LTP via a postsynaptic mechanism in amygdala and neocortex (see review [13]). This LTP does not require activation of NMDA receptors [40] or an increase of intracellular calcium [41]. However, LTP of inhibitory synapses in cerebellar Purkinje cells [42], visual cortex [43], the CA3 area of hippocampus [44] and in deep cerebellar nuclei [45] depends on a postsynaptic increase in intracellular calcium. Our results indicate that presynaptic NMDA receptors in the developing neocortex mediate a novel type of synaptic plasticity consisting of an early transient potentiation followed by a sustained increase in IPSC amplitude.

At most glutamatergic synapses, NR2B subunits are abundantly expressed at birth and are gradually replaced by NR2A subunits during development [46], [47]. NMDA receptors largely consist of NR1/NR2B subunits at newly formed synapses whereas at mature synapses NR2A-containing receptors are targeted to synapses. Homomeric NR2B receptors are kept from the postsynaptic membrane [48]. Consistent with this developmental scheme, we have shown that presynaptic NR2B-containing receptors modulate GABA release early in development (PND12-15) and appear to be lost at older ages (PND21-25). The high glutamate affinity of NR2B containing receptors [49] make them ideally suited for sensing glutamate released during patterned stimulation. It remains to be determined why presynaptic NMDA receptors are lost during this period where postsynaptic receptors successfully make the NR2B to NR2A switch. Numerous developmental studies have focused on the switch between NR2B to NR2A receptors. However, expression of NR3A containing NMDA receptors is also developmentally regulated [50]. Their low sensitivity to Mg^2+^ blockade would enable contribution to synaptic transmission under physiological conditions.

The concentration of extracellular glycine and D-serine was not controlled in the present experiments. Increasing the glycine or D-serine concentration did not change the frequency or peak amplitude of mEPSCs in immature rat visual cortex whereas D-APV decreased event frequency [51]. The observed apparent loss of presynaptic NMDA receptors at PND 21-25 could be due to developmental changes in glycine and or D-serine levels. It could also be attributable to a switch to receptors containing a subunit with a higher EC50 for glycine. In such a case, raising glycine concentrations could unmask presynaptic NMDA receptors in more mature animals.

### Regulation of release by presynaptic NMDA receptors

The physiological effects of presynaptic NMDA receptors reported here and by others in a variety of brain regions (see review [13]) are observed in the presence of physiological concentrations of extracellular Mg^2+^. The mechanisms whereby NMDA receptors could act to increase transmitter release under these conditions are unclear. It is well-established that NMDA receptors are blocked in a voltage-dependent manner by Mg^2+^. The resting potential in GABAergic synaptic terminals is largely unknown. Resting potentials of −60 to −85 mV have been reported for mossy fiber terminals [52] and at the Calyx of Held [53] suggesting that Mg^2+^ block would be present. The question is how much current flows through presynaptic NMDA receptors under normal conditions. We have previously shown that there are developmental changes in the voltage-dependent Mg^2+^ block of neocortical NMDA responses [54]. However, even in PND9-14 animals, substantial current flow was observed at −80 mV. Most central nerve terminals are small structures and are likely to have high input resistances; small NMDA currents could depolarize the nerve terminals sufficiently to relieve the Mg^2+^ block, particularly if the receptor density was high.

Enhancement of GABA release by presynaptic NMDA receptors presumably involves increases in terminal Ca^2+^ levels. This could be due to direct entry via NMDA receptors [18] or activation of voltage-gated calcium channels in response to depolarization [55]. Secondary release of calcium from intracellular stores, as reported for kainate receptor activation [22], is also a possible mechanism. Multiple actions of calcium on release mechanisms are then possible [13]. Alternatively, involvement of other retrograde messengers, released following activation of NMDA receptors on other neurons or glia, acting on GABAergic terminals remains a possibility [13].

### IPSP plasticity and network assembly

Information in the brain is believed to be encoded by large and overlapping networks of interconnected neurons. Neuronal plasticity, a process that depends on cellular activity and environmental experiences [56], fine-tunes neuronal connectivity. Although experiences shape neuronal networks to a limited extent in the adult brain, organization of local networks is more robust during early postnatal development. Tonic and phasic modulation of inhibitory activity by presynaptic NMDA receptors could be a significant mechanism contributing to circuit formation and may be important for proper functioning of local cortical networks during development.

In summary, our results indicate that presynaptic NMDA receptors modulate GABA release onto neocortical pyramidal cells in the early postnatal period (PND12-15). Ambient glutamate levels produce tonic facilitation of inhibitory synaptic transmission via activation of presynaptic NMDA receptors whereas trains of patterned stimulation induced IPSC LTP.

## Materials and Methods

### Ethics Statement

Animals were handled and housed according to the guidelines from the NIH Committee on Laboratory Animal Resources. All experimental protocols were approved by the University of Alabama Institutional Animal Care and Use Committee. Every effort was made to minimize pain and discomfort.

### Slice preparation

Slices of rat frontal association cortex were prepared from PND12-25 animals using procedures similar to those described previously [22]. Rats were anesthetized with ketamine (100 mg/kg) and decapitated. The brain was quickly removed and placed in ice-cold saline, containing (in mM): 125 NaCl, 3.5 KCl, 0.5 CaCl_2_, 3.5 MgCl_2_, 26 NaHCO_3_ and 10 D-glucose. The saline was bubbled with 95%O_2_/5%CO_2_ to maintain pH around 7.4. Coronal brain slices (300 µm thick), containing frontal association cortex were cut using a Vibratome (Ted Pella, Inc., Riverside, CA). The slices were kept for 30 minutes at 37°C and then stored at room temperature (22–23°C). Individual slices were transferred to a recording chamber mounted on the stage of a Leica DM LFSA (Leica Microsystems Wetzlar GMBH, Wetzlar, Germany) microscope equipped with infrared Nomarski differential interference contrast optics. The chamber was continuously perfused with oxygenated saline at a rate of 2–4 ml/min at 32°C. For whole-cell patch clamp recordings, individual cells were visualized using a Leica 40X, 0.75 NA long-working distance water immersion objective. Neurons were imaged using an infrared-sensitive video camera (Newvicon C2400-07-C, Hamamatsu, Japan) and displayed on a video monitor. Pyramidal neurons in layers II/III were identified by their depth below the pial surface, pyramidal shape, presence of a prominent apical dendrite and their regular spiking properties.

### Solutions and chemicals

The extracellular recording solution contained (in mM): 125 NaCl, 3.5 KCl, 1.25 NaH_2_PO_4_, 2.5 CaCl_2_, 1.3 MgSO_4_, 26 NaHCO_3_, and 10 D-glucose. The patch pipette solution had the following composition (in mM): 135 KCl, 0.5 ethyleneglycol-bis-(β-aminoethyl ether)-N,N,N',N',-tetraacetic acid (EGTA), 2 Mg-ATP, 0.2 NaGTP and 10 HEPES. The pH was adjusted to 7.3 with 1M KOH and osmolarity was adjusted to 290-300 mOsm with sucrose. CNQX, D-APV, GYKI52466, MK-801, Ro25-6981, TTX and SCH50911 were purchased from Tocris Cookson Inc. (Ellisville, MO).

### Stimulation

Synaptic responses were evoked with a bipolar stimulating electrode consisting of a twisted pair of 25 µm Formvar insulated nichrome wires. The electrode was positioned 150–200 µm below the recording pipette. Stimulation pulses were 50–200 µA in amplitude and 80–100 µs in duration. A series of increasing stimulations were applied until stable IPSCs were evoked. Paired stimulation at an interpulse interval of 50 ms and frequency of 0.05 Hz was then used. Physiologically patterned stimulation [25] was used to induce LTP. This consisted of 10 bursts at 1.25 Hz with each burst consisting of 10 stimuli at 25 Hz.

### Recording and analysis

Whole-cell patch-clamp recordings were obtained at 32°C. Patch pipettes were pulled from borosilicate glass capillaries on a Narishige Model PP-83 puller and had resistances of 3–4 MΩ. Whole-cell voltage-clamp recordings were made using an Axopatch 1B amplifier (Molecular Devices, Sunnyvale, CA), controlled by Clampex 8.0 software via a Digidata 1322A interface (Molecular Devices). Evoked responses were filtered at 2 kHz, digitized at 10 kHz and analyzed using Clampfit 8.0 software (Molecular Devices). Response amplitudes were measured as the difference between baseline and IPSC peak. Data are expressed as mean ± SEM. Statistical analysis of response amplitudes before, during, and after drug applications was carried out using a two-tailed Student's t-test with Statmost software (Data-Most). P<0.05 was considered significant.

## References

[1] Lo Turco JJ, Kriegstein AR (1991). Clusters of coupled neuroblasts in embryonic neocortex.. Science.

[2] Luhmann HJ, Prince DA (1991). Postnatal maturation of the GABAergic system in rat neocortex.. J Neurophysiol.

[3] Manent J, Represa A (2007). Neurotransmitters and brain maturation: early paracrine actions of GABA and glutamate modulate neuronal migration.. Neuroscientist.

[4] Hensch TK Fagiolini M, Mataga N, Stryker MP, Baekkeskov S (1998). Local GABA circuit control of experience-dependent plasticity in developing visual cortex.. Science.

[5] Wang DD, Kriegstein AR (2008). GABA regulates excitatory synapse formation in the neocortex via NMDA receptor activation.. J Neurosci.

[6] Gaiarsa JL, Caillard O, Ben-Ari Y (2002). Long-term plasticity at GABAergic and glycinergic synapses: mechanisms and functional significance.. Trends Neurosci.

[7] Petralia RS, Wang YX, Wenthold RJ (1994). The NMDA receptor subunits NR2A and NR2B show histological and ultrastructural localization patterns similar to those of NR1.. J Neurosci.

[8] Charton JP, Herkert M, Becker CM, Schroder H (1999). Cellular and subcellular localization of the 2B-subunit of the NMDA receptor in the adult rat telencephalon.. Br Res.

[9] Martin D, Bustos GA, Bowe MA, Bray SD, Nadler JV (1991). Autoreceptor regulation of glutamate and aspartate release from slices of the hippocampal CA1 area.. J Neurochem.

[10] Bustos G, Abarca J, Forray MI, Gysling K, Bradberry CW (1992). Regulation of excitatory amino acid release by N-methyl-D-aspartate receptors in rat striatum: in vivo microdialysis studies.. Br Res.

[11] Berretta N, Jones RSG (1996). Tonic facilitation of glutamate release by presynaptic N-methyl-D-aspartate autoreceptors in the entorhinal cortex.. Neuroscience.

[12] Woodhall G, Evans DI, Cunningham MO, Jones RSG (2001). NR2B-containing NMDA autoreceptors at synapses on entorhinal cortical neurons.. J Neurophysiol.

[13] Corlew R, Brasier DJ, Feldman DE, Philpot BD (2008). Presynaptic NMDA receptors: Newly appreciated roles in cortical synaptic function and plasticity.. Neuroscientist.

[14] Yang J, Woodhall GL, Jones RSG (2006). Tonic facilitation of glutamate release by presynaptic NR2B-containing NMDA receptors is increased in the entorhinal cortex of chronically epileptic rats.. J Neurosci.

[15] Corlew R, Wang Y, Ghermazien H, Erisir A, Philpot BD (2007). Developmental switch in the contribution of presynaptic and postsynaptic NMDA receptors to long-term depression.. J Neurosci.

[16] DeBiasi S, Minelli A, Melone M, Conti F (1996). Presynaptic NMDA receptors in the neocortex are both auto- and heteroreceptors.. NeuroReport.

[17] Paquet M, Smith Y (2000). Presynaptic NMDA receptor subunit immunoreactivity in GABAergic terminals in rat brain.. J Comp Neurol.

[18] Glitsch M, Marty A (1999). Presynaptic effects of NMDA in cerebellar purkinje cells and interneurons.. J Neurosci.

[19] Duguid IC, Smart TG (2004). Retrograde activation of presynaptic NMDA receptors enhances GABA release at cerebellar interneuron–Purkinje cell synapses.. Nat Neurosci.

[20] Liu SJ, Lachamp P (2006). The activation of excitatory glutamate receptors evokes a long-lasting increase in the release of GABA from cerebellar stellate cells.. J Neurosci.

[21] Nugent FS, Penick EC, Kauer JA (2007). Opiods block long-term potentiation of inhibitory synapses.. Nature.

[22] Mathew SS, Hablitz JJ (2008). Calcium release via activation of presynaptic IP3 receptors contributes to kainate-induced IPSC facilitation in rat neocortex.. Neuropharmacol.

[23] Sah P, Hestrin S, Nicoll RA (1989). Tonic activation of NMDA receptors by ambient glutamate enhances excitability of neurons.. Science.

[24] Varela JA, Song S, Turrigiano GG, Nelson SB (1999). Differential depression at excitatory and inhibitory synapse in visual cortex.. J Neurosci.

[25] Rose GM, Dunwiddie TV (1986). Induction of hippocampal long-term potentiation using physiologically patterned stimulation.. Neurosci Lett.

[26] Trommer BL, Kennelly JJ, Colley PA, Overstreet LS, Slater NT (1995). APV blocks LTP in developing rat dentate gyrus and unmasks LTD.. Exp Neurol.

[27] Sjöström PJ, Turrigiano GG, Nelson SB (2003). Neocortical LTD via coincident activation of presynaptic NMDA and cannabinoid receptors.. Neuron.

[28] Campbell SL, Hablitz JJ (2004). Glutamate transporters regulate excitability in local networks in rat neocortex.. Neuroscience.

[29] Campbell SL, Mathew SS, Hablitz JJ (2007). Pre- and postsynaptic effects of kainate on layer II/III pyramidal cells in rat neocortex.. Neuropharmacology.

[30] Featherstone D, Shippy DE (2008). Regulation of synaptic transmission by ambient extracellular glutamate.. Neuroscientist.

[31] Herman MA, Jahr CE (2007). Extracellular glutamate concentration in hippocampal slice.. J Neurosci.

[32] Bandrowski AE, Huguenard JR, Prince DA (2003). Baseline glutamate levels affect group I and II mGluRs in layer V pyramidal neurons of rat sensorimotor cortex.. J Neurophysiol.

[33] Christie JM, Jahr CE (2008). Dendritic NMDA receptors activate axonal calcium channels.. Neuron.

[34] Christie JM, Jahr CE (2009). Selective expression of ligand-gated ion channels in L5 pyramidal cell axons.. J Neurosci.

[35] Kamiya H, Ozawa S (2000). Kainate receptor-mediated presynaptic inhibition at the mouse hippocampal mossy fibre synapse.. Journal of Physiology (Lond).

[36] Stell BM, Mody I (2002). Receptors with different affinities mediate phasic and tonic GABA_A_ conductances in hippocampal neurons.. J Neurosci.

[37] Deisz RA, Prince DA (1989). Frequency-dependent depression of inhibition in guinea-pig neocortex in vitro by GABA_B_ receptor feed-back on GABA release.. J Physiol (Lond).

[38] Axmacher N, Winterer J, Stanton PK, Draguhn A, Muller W (2004). Two-photon imaging of spontaneous vesicular release in acute brain slices and its modulation by presynaptic GABA_A_ receptors.. NeuroImage.

[39] Xie Z, Sastry BR (1991). Inhibition of protein kinase activity enhances long-term potentiation of hippocampal IPSPs.. NeuroReport.

[40] Shew T, Yip S, Sastry BR (2000). Mechanisms involved in tetanus-induced potentiation of fast IPSCs in rat hippocampal CA1 neurons.. J Neurophysiol.

[41] Morishita W, Sastry BR (1991). Chelation of postsynaptic Ca2+ facilitates long-term potentiation of hippocampal IPSPs.. NeuroReport.

[42] Kano M, Rexhausen U, Dreesen J, Konnerth A (1992). Synaptic excitation produces a long-lasting rebound potentiation of inhibitory synaptic signals in cerebellar purkinje cells.. Nature.

[43] Komatsu Y, Iwakiri M (1993). Long-term modification of inhibitory synaptic transmission in developing visual cortex.. NeuroReport.

[44] McLean HA, Caillard O, Ben-Ari Y, Gaiarsa JL (1996). Bidirectional plasticity expressed by GABAergic synapses in the neonatal rat hippocampus.. J Physiol (Lond).

[45] Aizenman CD, Manis PB, Linden DJ (1998). Polarity of long-term synaptic gain change is related to postsynaptic spike firing at a cerebellar inhibitory synapse.. Neuron.

[46] Carmignoto G, Vicini S (1992). Activity-dependent decrease in NMDA receptor responses during development of the visual cortex.. Science.

[47] Hestrin S (1992). Developmental regulation of NMDA receptor-mediated synaptic currents at a central synapse.. Nature.

[48] Tovar KR, Westbrook GL (1999). The incorporation of NMDA receptors with a distinct subunit composition at nascent hippocampal synapses *in vitro*.. J Neurosci.

[49] Priestley T, Laughton P, Myers J, Le Bourdelles B, Kerby J (1995). Pharmacological properties of recombinant human N-methyl-D-aspartate receptors comprising NR1a/NR2A and NR1a/NR2B subunit assemblies expressed in permanently transfected mouse fibroblast cells.. Mol Pharmacol.

[50] Roberts AC, Diez-Garcia J, Rodriguiz RM, Lopez IP, Lujan R (2009). Downregulation of NR3A-Containing NMDARs Is Required for Synapse Maturation and Memory Consolidation.. Neuron.

[51] Li Y-H Han T-Z (2006). Glycine binding sites of presynaptic NMDA receptors may tonically regulate glutamate release in the rat visual cortex.. J Neurophysiol.

[52] Geiger JRP, Jonas P (2000). Dynamic control of presynaptic Ca^2+^ inflow by fast-inactivating K^+^ channels in hippocampal mossy fiber boutons.. Neuron.

[53] Forsythe ID (1994). Direct patch recording from identified presynaptic terminals mediating glutamatergic EPSCs in the rat CNS, in vitro.. J Physiol (Lond).

[54] Burgard EC, Hablitz JJ (1994). Developmental changes in the voltage-dependence of neocortical NMDA responses.. Dev Br Res.

[55] Awatramani GB, Price GD, Trussell LO (2005). Modulation of transmitter release by presynaptic resting potential and background calcium levels.. Neuron.

[56] Katz LC, Shatz CJ (1996). Synaptic activity and the construction of cortical circuits.. Science.

